# Right-lateralized alpha desynchronization during regularity discrimination: Hemispheric specialization or directed spatial attention?

**DOI:** 10.1111/psyp.12399

**Published:** 2014-12-23

**Authors:** Damien Wright, Alexis D J Makin, Marco Bertamini

**Affiliations:** Department of Psychological Sciences, University of LiverpoolLiverpool, UK

**Keywords:** Symmetry, Alpha, Sustained posterior negativity, Event-related desynchronization, Lateralization

## Abstract

When actively classifying abstract patterns according to their regularity, alpha desynchronization (ERD) becomes right lateralized over posterior brain areas. This could reflect temporary enhancement of contralateral visual inputs and specifically a shift of attention to the left, or right hemisphere specialization for regularity discrimination. This study tested these competing hypotheses. Twenty-four participants discriminated between dot patterns containing a reflection or a translation. The direction of the transformation, which matched one half onto the other half, was either vertical or horizontal. The strategy of shifting attention to one side of the patterns would not produce lateralized ERD in the horizontal condition. However, right-lateralized ERD was found in all conditions, regardless of orientation. We conclude that right hemisphere networks that incorporate the early posterior regions are specialized for regularity discrimination.

Natural processes often produce emergent symmetry, which can be seen in countless examples from crystals, to galaxies, to animal phenotypes (Tyler, 1995). Psychophysical studies have shown that reflectional symmetry is more salient, and more easily detected, by the human visual system than other regularities, such as translation or rotation (Bertamini, 2010; Julesz, 1971; Koning & Wagemans, 2009), despite the fact that these patterns all share the presence of a rigid transformation (Mach, 1886/1959; Makin, Pecchinenda, & Bertamini, 2012). Reflectional symmetry is particularly salient when the axis of reflection is vertical (Barlow & Reeves, 1979). Sensitivity to reflection could be adaptive because reflectional symmetry signals reproductive fitness in potential mates (Moller, 1992; Rhodes, Proffitt, Grady, & Sumich, 1998), or because it is often a property of whole objects and therefore plays a role in image segmentation and object identification (Pizlo & Stevenson, 1999).

Symmetry refers to the property of a stimulus, which is defined as a geometric invariance under a rigid transformation such as reflection, rotation, or translation. Therefore, multiple symmetries can be present in a stimulus, and in the case of reflection there may be single or multiple axes. In this experiment, when we refer to symmetry we are concerned with the rigid transformations, which include reflection, translation, and rotation. When we discuss symmetry discrimination, we mean discrimination between two different transformations, here, reflection and translation.

The neuroimaging literature on symmetry has reported activations in a number of areas including the lateral occipital complex (LOC), V3a, V4, and V7, but not in the primary or secondary visual cortices (Chen, Kao, & Tyler, 2007; Sasaki, Vanduffel, Knutsen, Tyler, & Tootell, 2005; Tyler et al., 2005). Transcranial magnetic stimulation studies have largely corroborated these results. Cattaneo, Mattavelli, Papagno, Herbert, and Silvanto (2011) found that adaptation to symmetry was altered by disruption of either left or right LOC; however, no such effect was produced by V1 disruption. More recently, Bona, Herbert, Toneatto, Silvanto, and Cattaneo (2014) showed that TMS disruption of either left or right LOC impaired symmetry discrimination, but the effect was stronger on the right. We examine the issue of right lateralization with a different technique in the current work.

Several studies have used ERPs to study symmetry perception. Norcia, Candy, Pettet, Vildavski, and Tyler (2002) presented participants with reflection or random patterns in quick succession. Amplitude in posterior electrodes was more negative for symmetrical patterns after around 220 ms from stimulus onset. Jacobsen and Höfel (2003) measured ERPs while participants judged abstract patterns as symmetrical or random. Again, amplitude at posterior electrodes was relatively negative for symmetrical patterns for a prolonged period after the visual evoked potential. They termed this component the sustained posterior negativity (SPN). The SPN was recorded in subsequent experiments when participants were engaged in oddball detection rather than symmetry discrimination (Höfel & Jacobsen, 2007a) or when participants were deliberately misreporting their responses (Höfel & Jacobsen, 2007b). Makin, Wilton, Pecchinenda, and Bertamini (2012) recorded the SPN, and found that it was unaffected by whether reflection or random patterns were designated as targets in their two-alternative forced choice discrimination task. Makin, Rampone, Pecchinenda, and Bertamini (2013) reported an SPN for different regularities, although reflection produced the largest response. Finally, Makin, Rampone, Wright, Martinovic, and Bertamini (2014) found that the SPN was larger for reflection than translation, independently of the requirements of the discrimination task, and independently of whether the regularity was the property of a single object or the gap between two objects. So far, it seems reasonable to conclude that the SPN is generated by automatic visual symmetry analysis in the extrastriate visual cortex, and this activity seems to systematically map onto some, but not all, psychophysical findings.

Makin, Wilton et al. (2012) also analyzed their EEG data in another way, measuring event-related desynchronization (ERD) of the occipital alpha rhythm. This response is fundamentally different to the SPN. ERD was comparable for reflection and random trials, and was significantly greater over the right posterior region. Makin et al. (2014) replicated this right lateralization, and found that it was only present when participants were actively discriminating regularity (reflection or translation) and not when they were discriminating the number of objects in the display (one or two), even though the visual stimuli were identical in both tasks. It seems that alpha ERD picks up a different aspect of visual symmetry perception to the SPN: The SPN is the neural response to symmetry—it is a difference wave that distinguishes symmetry from random, and between different types of symmetry. Regularity detectors generate the SPN. Conversely, posterior alpha ERD is the same for all regularities and for random patterns. It is right lateralized, across all conditions, but only when people are engaged in a symmetry discrimination task. Right lateralization of posterior alpha ERD is thus a correlate of engagement with a task about regularity rather than regularity detection.

For many years, alpha oscillations have been associated with cortical off states. For example, alpha power is greater with the eyes closed, or when participants are not engaging in a task (Pfurtscheller & Lopes da Silva, 1999). Attention has also been shown to modulate alpha rhythms: with a decrease in alpha and an increase in beta power during attentional tasks (Gómez, Vázquez, Vaquero, López-Mendoza, & Cardoso, 1998; Vázquez, Gómez, Vaquero, & Cardoso, 2001). According to the inhibition-timing hypothesis, synchronized alpha oscillations (∼8–12 Hz) reflect top down inhibition rather than purely “cortical idling.” Conversely, a reduction in alpha power, desynchronization, reflects neural excitation produced by task engagement (Klimesch, Sauseng, & Hanslmayr, 2007). The right-lateralized alpha response probably arises from greater activation in the posterior right hemisphere compared to the left during regularity discrimination tasks. However, these findings are inconclusive, because lateralization could arise from either transitory enhancement of contralateral visual inputs, or from functional differences between the cerebral hemispheres.

In our previous work, the axis of orientation was always vertical (Makin et al., 2014; Makin, Wilton et al., 2012). This may have encouraged participants to explore the regularity by shifting attention back and forth across the midline. Although eye movements were suppressed in these experiments, participants may still have moved covert attention. It is conceivable that visual exploration begins with a systematic shift to the left after early visual processing, and that this manifests as right-sided alpha desynchronization. Alternatively, there may be genuine hemispheric differences in regularity processing, with more regularity sensitive systems in the right posterior regions.

This later hypothesis is plausible because of the differences in cognitive functions of the two hemispheres. The exact nature of hemispheric specialization is still debated, but important differences have been suggested. Beyond the well-established left specialization for language and right specialization for spatial processing (Cai, Van der Haegen, & Brysbaert, 2013), it has been proposed that the left hemisphere preferentially processes high spatial frequencies whereas the right hemisphere preferentially processes low spatial frequencies (Sergent, 1983). In addition, the left hemisphere may be involved in processing local elements whereas the right is more involved in global element processing (Van Kleeck, 1989). Finally, there is strong evidence that the right frontoparietal network is specialized for mental object rotation (Parsons, 2003) and directing of visuospatial attention (Mesulam, 2002).

Most relevantly for the current study, there is some evidence for right hemisphere specialization for symmetry detection. First, Corballis and Roldan (1974) found that symmetrical patterns could be detected slightly faster when presented to the left visual hemifield (i.e., processed by the right hemisphere), and Brysbaert (1994) replicated this modest effect. Wilkinson and Halligan (2002) considered the similarities between symmetry perception and line bisection (where people place a mark in the center of a horizontal line, or attempt to identify noncentral bisections). A right hemisphere advantage was found for both tasks. Stronger evidence for right hemisphere dominance in symmetry detection comes from a recent study by Verma, Van der Haegen, and Brysbaert (2013), who briefly presented symmetrical or asymmetrical block shapes to either hemisphere while participants fixated centrally. For the neuro-typical participants who were left hemisphere dominant for language, symmetry detection was superior when images were presented to the right hemisphere. For a subgroup of unusual right hemisphere language participants, this bias was absent or sometimes reversed. In short, it is likely symmetry detection systems are present in both cerebral hemispheres, but that the right hemisphere dominates in most people. However, the existing literature documents right hemisphere advantage when reflection symmetry is presented, not when random or translation patterns are presented. This is different from the right-lateralized ERD response found by Makin, Wilton et al. (2012), which was equivalent during symmetrical and random presentations. What was critical in the ERD work was that observers were engaged in a symmetry discrimination task.

In this study, participants saw reflection or translation patterns, while EEG responses were recorded. The orientation of the pattern was either horizontal or vertical (Figure 1). In the case of reflection, this means a vertical or horizontal axis of symmetry, but in both cases (reflection and translation) a rigid transformation matches elements in one half of the stimulus to elements in the other half. Therefore, vertical and horizontal orientation refers to the separation between these two halves.

**Figure 1:**
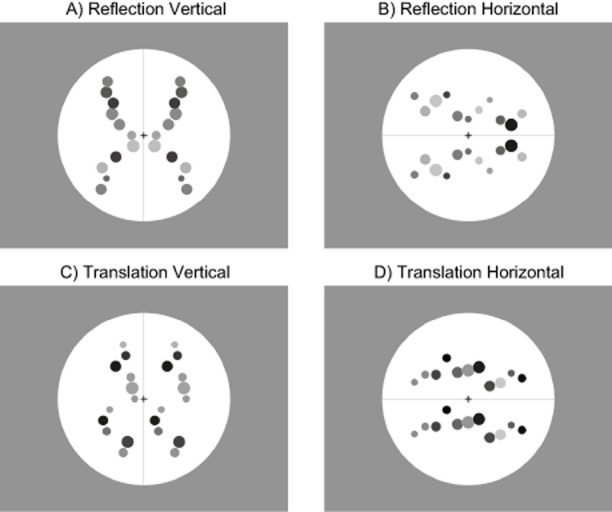
Example stimuli from the four conditions (vertical reflection, horizontal reflection, vertical translation, and horizontal translation). Actual stimuli were generated so as to be different in each trial. Participants discriminated reflection from translation.

A “look left” strategy predicts that ERD lateralization should only occur in the vertical condition. In the horizontal condition, the same strategy would involve moving attention up and down, rather than left and right, and this would not result in systematically right-lateralized ERD. Conversely the right hemisphere specialization hypothesis predicts comparable lateralized ERD in horizontal and vertical conditions. There is potential for confusion here: To reiterate a point made above, posterior ERD is expected to be equivalent on reflection and translation trials (as found by Makin et al., 2014). The novel question in this work was whether this ubiquitous right lateralization during regularity discrimination would be observed when the patterns are horizontally orientated.

A secondary aim of this study was to investigate the role of orientation on the symmetry-related ERPs, which has not been studied extensively. Some psychophysical experiments have found that the vertical axis of reflectional symmetry is more salient than the horizontal axis (e.g., Friedenberg & Bertamini, 2000). It is expected that there will be a larger SPN in the vertical condition than the horizontal condition. This would be consistent with the findings of Makin et al. (2013), who found a relationship between visual salience and SPN amplitude.

## Method

### Participants

Twenty-four participants took part in the study (age 18–44, mean age 22, 6 males, 1 left-handed). Participants had normal or corrected-to-normal vision, and some received course credit upon completion of the study. The study was approved by the Ethics Committee of the University of Liverpool and conducted in accordance with the Declaration of Helsinki (revised 2008).

### Apparatus

Participants sat 100 cm from the monitor (1,280 × 1,024; 60 Hz, Mitsubishi, Tokyo, Japan) with their head stabilized with a chin rest. Participants used the A and L buttons of the computer keyboard to enter their responses. Stimuli were presented on a CRT monitor and controlled with open source PsychoPy software (Peirce, 2007). EEG activity was recorded using a BioSemi (Amsterdam, The Netherlands) Active-Two amplifier in an electrically shielded and darkened room. EEG was sampled continuously at 512 Hz from 64 scalp electrodes arranged according to the standard International 10–20 system. Common mode sense (CMS) and driven right leg (DRL) were used as reference and ground electrodes. Vertical bipolar electrodes (VEOG) were positioned above and below the right eye. Horizontal bipolar electrodes (HEOG) electrodes were positioned on the outer canthi of both eyes. These were used to detect blinks and eye movements.

### Design

The study had a within-subjects design: Regularity (reflection, translation) × Orientation (horizontal, vertical) with 72 trials per condition. The trials were presented in a randomized sequence for each participant.

### Stimuli

Stimuli consisted of filled gray circles that varied in brightness (Figure 1). In each half of the patterns there were 11 elements, which varied in radius between 0.5° and 1°. There were 0.9° between the centers of the dots. The patterns were presented either with a vertical or a horizontal orientation with a line going through the center of the pattern indicating the orientation. A black fixation cross also appeared at the center of each pattern. The background consisted of a white circle, which had a diameter of 14.4°. Vertical patterns were very similar to those used by Makin et al. (2013).

### Procedure

Participants sat in front of a CRT monitor in a darkened and electrically shielded room. The experiment consisted of a total of 288 trials. Each trial began with a 1.5-s baseline period, when the screen showed the background circle, the central fixation cross, and the oriented line. The dot elements then appeared reflected or translated on either side of the midline. The stimuli stayed on screen for 2 s. This design ensured that axis orientation was predictable before presentation, and thus participants did not have to compute this while making reflection-translation judgments. This ensured a cleaner measure of the neural response to the different regularities than would have been possible if orientation was unpredictable before stimulus onset. With this design, it made sure that the time to perceive the orientation did not vary between the reflection and the translation conditions.

After each trial, participants were presented with a response screen, and they had to report whether the observed pattern was a reflection or a translation. The response screen informed them to press the button on the left for “reflection” and on the right for “translation” or vice versa. The two orders varied between the trials and were counterbalanced across conditions so that no motor planning was possible before the response screen appeared (Makin, Wilton et al., 2012). Participants had up to 10 s to log a response. The experiment was divided into eight blocks, which allowed participants to have breaks in which they could rest their eyes.

Prior to the start of the main experiment, participants completed a practice block. This consisted of eight trials, and its design reflected that of the main experiment.

### EEG Analysis

EEG data was processed using the EEGLAB toolbox in MATLAB (Delorme & Makeig, 2004). The raw EEG signals from the 64 electrodes were rereferenced offline to a scalp average and low-pass filtered at 40 Hz. The data were then sampled at 128 Hz in order to reduce file size and segmented into −1-s to 2-s epochs with a baseline of −200 ms to 0 ms. Ocular and muscle artifacts were identified and removed using independent components analysis (ICA). The data were then re-formed as 64 independent components and an average of 11.4 components removed from each participant (min = 1, max = 18). After ICA, trials that had amplitude greater than ± 100 μV for any electrode were removed. The average proportion of excluded trials did not differ significantly between the four conditions (reflection vertical, 18%; translation vertical, 15%; reflection horizontal, 17%; translation horizontal, 14%, *F*(3,69) = 2.475, *p* = .069, 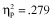).

Time frequency analysis was performed on the same cleaned data that were used for the ERP analysis, using the FieldTrip toolbox for MATLAB (Oostenveld, Fries, Maris, & Schoffelen, 2011). Frequency bands from 5 to 20 Hz were explored, with a −500 to 0 ms baseline. Raw data were convolved with a Hanning-tapered wavelet comprising four cycles at each frequency. Relative power was then computed as a proportion change from baseline. Wavelets were positioned at increments separated by 50 ms through the raw data. This means that low frequency wavelets overlapped to a greater degree than high frequency ones. The preprocessing steps were matched with Makin et al. (2014). We measured desynchronization in the 10–14 Hz frequency band from 400 to 1,000 ms poststimulus onset. These parameters were similar, but not identical, to those used by Makin et al. (2014), that is, 400–700 ms, 8–13 Hz, where right lateralized alpha ERD was also measured during reflection translations. The time-frequency window used by Makin et al. (2014) was not centered on the effects here, so the parameters were adjusted. This decision did not substantially affect the results. Secondary analysis reported in the online supporting information showed essentially the same ERD effects when the same window as Makin et al. (2014) was used.

### Electrooculogram Analysis

Although participants were instructed to fixate and eye movement artifacts were removed, these measures are not perfect. Therefore, it was important to establish whether eye movements and blinks contaminated some conditions more than others. To do this, the electrooculogram (EOG) analysis techniques used in our previous studies were improved (e.g., Makin et al., 2013; Makin, Wilton et al., 2012) by measuring EOG activity at the time window of the SPN or ERD, and only for trials included in the ERP and ERD analysis. For the selected EOG data, we computed the difference between maximum and minimum amplitude, then averaged this metric over all trials in each condition.

VEOG activity from the SPN window (250 to 1,000 ms) was analyzed with repeated measures analysis of variance (ANOVA): Regularity (reflection, translation) × Orientation (vertical, horizontal). Ideally, there would have been no effects or interactions; however, there was significantly more VEOG activity in the reflection trials than the translation trials, *F*(1,23) = 10.03, *p* = .004, 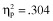, and in the vertical trials than the horizontal trials, *F*(1,23) = 5.77, *p* = .025, 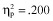. There was no Regularity × Orientation interaction, *F*(1,23) < 1, *n.s*. This pattern differs from SPN results reported below. Next, the same analysis was performed, but using VEOG activity from the time window used for posterior ERD (400 to 1,000 ms). There were main effects of regularity, *F*(1,23) = 11.43, *p* = .003, 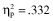, and orientation, *F*(1,23) = 5.46, *p* = .029, 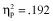, and no interaction, *F*(1,23) < 1, *n.s*. Again, this is a different pattern from the ERD results reported below.

To further establish that differential blinking was not responsible for posterior ERPs, potential correlations between the VEOG metric and amplitude at bilateral posterior electrode clusters were measured. There was no significant correlation in any of the four conditions (maximum *r* = .24, *p* = .268). Next, similar correlations between VEOG activity and bilateral occipital alpha ERD were examined, and there were no significant correlations here either (maximum *r* = −.34, *p* = .105). Finally, there were no correlations between right lateralization of posterior ERD and VEOG activity (maximum *r* = .16, *p* = .442). It can be concluded that the effects of interest recorded at posterior electrodes do not reflect differential blinking.

Next, the same analysis of HEOG data from the SPN window (250 to 1,000 ms) was conducted. There were no effects or interactions, *F*(1,23) < 1, *n.s*. Furthermore, there were no effects when the ERD window was examined (400 to 1,000 ms; *F*(1,23) < 1, *n.s*.). This shows that unwanted horizontal eye movements were equally distributed across conditions, and thus do not explain the effects of interest.

There were no correlations between posterior ERP amplitude and HEOG metric (maximum *r* = −.32, *p* = .131). There was no correlation between HEOG and the bilateral ERD response in any condition (maximum *r* = −.12, *p* = .592), and no correlations between HEOG and ERD lateralization (maximum *r* = −.15, *p* = .470).

In summary, there were some differences in VEOG activity between conditions, while unwanted HEOG activity was equally prevalent across conditions. Moreover, very little variance in the effects of interest was explained by individual variability of the EOG metrics. It can be concluded that the results reported below cannot be attributed to gross eye movement artifacts. Further examination of this issue is reported below.

## Results

### Behavioral Results

Participants discriminated patterns as reflection or translation. They made a correct discrimination on most of the trials (mean correct = 97.04%), with no differences between conditions (reflection, 97%; translation, 97%; horizontal, 97% vertical; 98%). Responses were entered after the patterns disappeared, and were unspeeded. Response times were not instructive in this study.

### Event-Related Potentials

**Figure 2:**
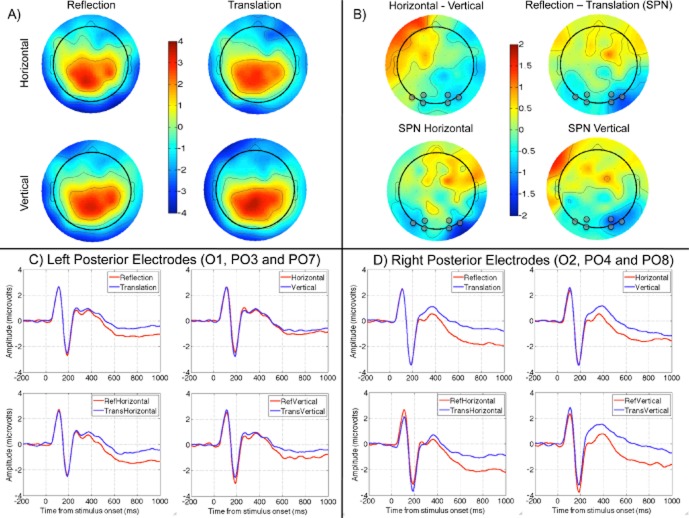
Event-related potentials. A: Grand-average topographic maps from the four conditions (vertical reflection, horizontal reflection, vertical translation, and horizontal translation) averaged over the 250–1,000 ms time window. B: Difference plots derived from this data. Electrodes used for analysis are highlighted with a gray dot. C: Grand-average ERP waves from left posterior electrodes (O1, PO3, and PO7) in different conditions. D: Equivalent data from right posterior electrodes (O2, PO4, and PO8).

Figure 2A shows topographic maps of grand-average ERPs from 250 to 1,000 ms. It can be seen that distribution of scalp activity was broadly comparable in the four conditions; however, difference maps, shown in Figure 2B, highlight important effects. There was an unexpected difference between horizontal and vertical trials, shown in the top left map. There was a clear SPN (i.e., amplitude was lower in reflection than the translation conditions), shown in the top right map. The SPN was present in both vertical and horizontal trials, as shown in the topographic maps below. It can be seen that SPN was larger on the right. Based on these difference plots, electrodes were selected for statistical analysis. These were O1, PO3, and PO7 and right-sided homologues, O2, PO4, and PO8. These electrodes are highlighted in gray in Figure 2B, and ERP waves from these electrodes are shown in Figures 2C, D (see supporting information for complementary analysis of SPN using different electrodes).

Amplitude in the 250 to 1,000 ms window was explored with repeated measures ANOVA: Hemisphere (left, right), × Regularity (reflection, translation) × Orientation (horizontal, vertical). As expected, there was a main effect for regularity, *F*(1,23) = 18.85, *p* < .001, 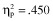, because amplitude was lower in reflection than translation trials. The only other significant effect was Regularity × Hemisphere interaction, *F*(1,23) = 5.26, *p* = .031, 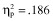. To explore this interaction, we analyzed left and right electrode clusters separately. The effect of regularity was significant in both clusters, but smaller on the left (left electrodes, *F*(1,23) = 9.47, p = .005, 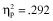; right electrodes, *F*(1,23) = 17.63, *p* < .001, 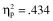. There were no significant effects involving orientation in the main analysis, although there was a borderline Hemisphere × Orientation interaction, *F*(1,23) = 3.98, *p* = .058, 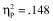. As suggested by Figure 2, there was an effect of orientation on the right, *F*(1,23) = 7.43, *p* = .012, 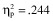, but not on the left, *F*(1,23) < 1, *n.s*.

### Time Frequency Analysis

**Figure 3:**
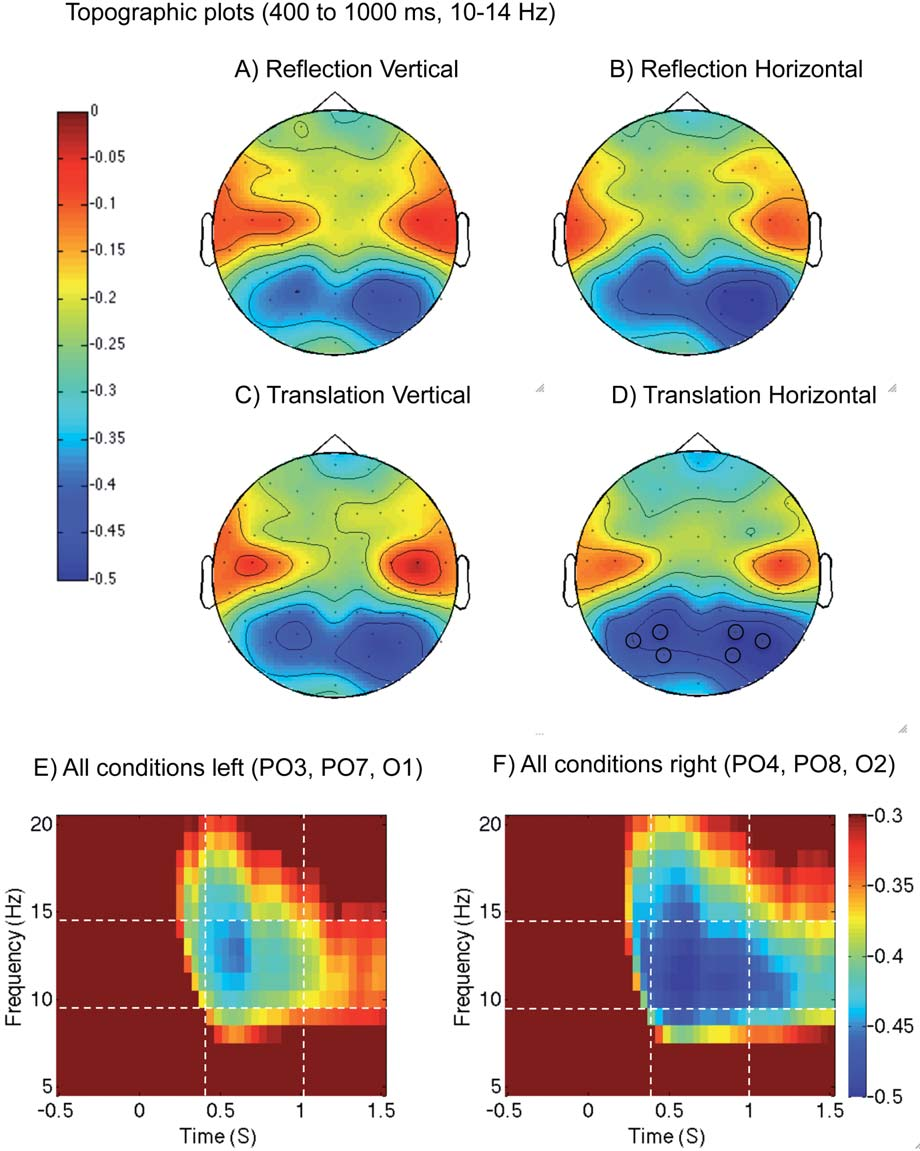
Event-related desynchronization. A–D: Scalp distribution of 10–14 Hz powers from 400 to 1,000 ms poststimulus onset. The analysis focused on posterior desynchronization (blue on these figures). Note that this response is bilateral, but stronger in the right hemisphere in all conditions. Electrodes used for analysis are highlighted with a gray dot. E, F: Time frequency spectrograms from left and right posterior electrode clusters, respectively (collapsed across all conditions). Note that ERD is greater on the right. Power is shown as proportion of power in the baseline interval (−500 to 0 ms). Dashed white lines indicate the time and frequency bands that were used to produce the topographic plots above and for statistical analysis.

Time frequency analysis is shown in Figure 3. The results were straightforward. At posterior electrodes, there was clear desynchronization in the 10–14 Hz band from around 400 ms onwards in all conditions (see supporting information for complementary analysis of different time windows and frequency bands). This ERD was more pronounced on the right hemisphere than the left in all conditions, and also stronger in horizontal than vertical trials. Baseline-relative alpha power was obtained in a set of left and right posterior electrodes where the effect was most pronounced (O1, PO3, and PO7 and right-sided homologues). Power was explored with three-factor repeated measures ANOVA: Hemisphere (left, right) × Regularity (reflection, translation) × Orientation (horizontal, vertical). There was a main effect of hemisphere, *F*(1,23) = 8.08, *p* = .009, 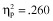, and orientation, *F*(1,23) = 12.434, *p* = .002, 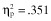, but no other effects or interactions (next largest effect regularity, *F*(1,23) = 3.289, *p* = .083, 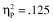, because posterior ERD was marginally larger for translation).

To get a sense of whether the right lateralization was driven by a small subgroup of participants, we tested the presence or absence of the effect in each participant (averaged across all four conditions). Seventeen of the 24 participants showed more alpha ERD in the right posterior electrodes (71%, *p* = .032, one-tailed binomial test).

## General Discussion

In previous work, Makin, Wilton et al. (2012) recorded posterior alpha desynchronization when people discriminated pattern regularity. This ubiquitous neural response to visual onsets indicates cortical excitation in posterior regions (Buzsáki, 2006; Klimesch et al., 2007). This ERD is evident over both hemispheres, but it was consistently stronger in right posterior electrodes (Makin et al., 2014; Makin, Wilton et al., 2012). However, this right lateralization in our previous work was inconclusive. It could result from either (a) a transitory shift of spatial attention to the left side of the patterns, enhancing contralateral inputs; or (b) a functional and anatomical specialization whereby the right posterior regions are more active during regularity discrimination.

In the current work, equivalent right lateralization of posterior alpha ERD was found when patterns were either vertically or horizontally oriented. Moving attention across the midline axis in the horizontal condition would involve moving attention upwards or downwards, which would not alter the balance of activity between left and right hemispheres. Therefore, right lateralization in the horizontal condition may have a different explanation. It is proposed that the right posterior regions are specialized for regularity discrimination, and are thus more active than the equivalent left hemisphere regions.

Despite the robust results, one cannot fully discard the look left hypothesis. It could be that participants visually explore the patterns by moving covert attention to the left hemifield, even in the horizontal condition. A leftward perceptual bias is commonly reported in judgments of magnitude, numerosity, and grayscale discrimination (Nicholls, Bradshaw, & Mattingley, 1999); this has been reported to be the consequence of an attentional bias (Nicholls & Roberts, 2002). It remains possible that this ubiquitous shift of spatial attention to the left could explain our current results. However, it is unlikely that the effect that we have measured results from a generic scanning bias because it was not present when observers did not engage in a symmetry discrimination task (Makin et al., 2014).

There may be functions of the right hemisphere that are activated during all tasks, and have nothing to do with the processing of reflection/translation. For example, the simple need to maintain fixation and generally engage attention may produce greater right hemisphere activation. To counter this, we refer again to Makin et al. (2014), who included a matched control condition where right lateralization was not apparent. Although further control experiments are required, there is important converging evidence from Bona et al. (2014), who found that TMS disruption of the right LOC had a greater effect on symmetry discrimination than the TMS disruption of the left LOC. We thus think it is likely that dedicated symmetry discrimination networks are right lateralized, and alpha ERD indexes this.

The current work can be related to previous findings on hemispheric specialization. The two best replicated findings on hemispheric specialization in humans are left lateralization for language, and right lateralization for spatial tasks. These biases may be causally related, and can be mutually reversed in some people (often left-handers, Cai et al., 2013). Regularity discrimination may be one kind of right hemisphere spatial task. Wilkinson and Halligan (2002) note that line bisection tasks require placing a mark at the center of a line, thus producing a symmetrical image. This ability is dramatically disrupted by right hemisphere damage compared to left hemisphere damage. These authors suggest that, while both hemispheres are sensitive to symmetry, there is right hemisphere specialization. In their Experiment 2, participants were faster and more accurate to detect symmetry when stimuli were flashed in the left visual field (i.e., processed by the right hemisphere). Moreover, in a recent study, Verma et al. (2013) found a similar left visual field advantage for symmetry detection in participants who were left lateralized for language (irrespective of handedness).

Although it is tempting to conclude that ERD lateralization is a simple manifestation of this apparent right brain specialization for symmetry perception, it is important to note that the ERD lateralization was comparable for both reflection and translation in this study, and in the findings of Makin et al. (2014). Moreover, in previous work right lateralization of alpha ERD was found for both reflection and random patterns (Makin, Wilton et al., 2012). Right-lateralized ERD is not a neural response to the presence of symmetry, but a signature of engagement with regularity discrimination tasks. The ERD in this study thus differs in an important way from the results of Wilkinson and Halligan (2002) and Verma et al. (2013), who found no hemispheric advantages when people responded to random stimuli.

Which right-lateralized brain networks display reduced alpha rhythm during all trials of a regularity discrimination task? It is thought that the occipital alpha rhythm is generated by excitation–inhibition cycles between visual cortical regions and the thalamus (e.g., Buzsáki, 2006). It is likely that the current work measured changes in oscillatory activity in visual areas, although these are, of course, subject to influences from higher brain regions (Laufs et al., 2006). However, there is some ambiguity here, which should not be glossed over: Most classic “right hemisphere dominant” functions, such as mental object rotation and spatial attention, are mediated by the parietal lobes, that is, well beyond the early visual maps that supposedly produce the posterior alpha rhythm. It could be that this experiment recorded ERD in the parietal regions rather than earlier visual regions, which is not so well documented. Alternatively, the posterior ERD could occur in early visual areas, but this could have been affected by ipsilateral top-down connections from functionally asymmetrical parietal areas. The current work cannot resolve such questions about the source of the scalp recordings.

If regularity discrimination mechanisms are right lateralized, one might expect to see converging evidence from fMRI studies. Jacobsen, Schubotz, Höfel, and Cramon (2006) compared activations produced by a discriminate symmetry task (collapsing over symmetry or random trials) with all conditions of an aesthetic judgment task (beautiful or ugly) and a control condition where participants made a trivial visual discrimination (arrow pointing left or right). Our results imply that there would be right-lateralized activity in the posterior regions during the discriminate symmetry task; however, Jacobsen et al. (2006) did not find this. As well as various frontal and parietal activations, the extrastriate visual cortex was found to be more active in the discriminate symmetry than in the control condition, while the left extrastriate visual cortex was more active during the discriminate symmetry task than the aesthetic judgment task. However, these fMRI results depend on the nature of the comparison tasks as much as the nature of the symmetry discrimination task. Right lateralization of alpha ERD is a reliable signature of regularity discrimination, although it is currently difficult to relate this to existing neuroimaging work on this topic, which has not reliably shown greater right hemisphere activation (Chen et al., 2007; Jacobsen et al., 2006; Sasaki et al., 2005; Tyler et al., 2005). Previous studies that have examined EEG and fMRI activity have shown that decreased alpha power correlates with increase blood-oxygen-level dependent (BOLD) signals in occipital regions (e.g., Goldman, Stern, Engel, & Cohen, 2002), so right lateralization of the BOLD signal would be expected. This has not been reported, although this may reflect differences in the nature of the signal and the tasks used. TMS studies have also failed to find consistent right lateralization, with one study finding a right hemisphere lateralization (Bona et al., 2014) while another did not (Cattaneo et al., 2011).

We found that horizontal patterns resulted in more occipital alpha ERD than vertical patterns. This effect was bilateral, implying more activation of both left and right posterior regions during horizontal trials. Previous symmetry perception research has shown that vertical orientations are detected faster (Friedenberg & Bertamini, 2000; Julesz, 1971). However, it is not clear whether the vertical advantage survives when axis orientation can be anticipated (Wenderoth, 1994; Wenderoth & Welsh, 1998). In this study, the orientation of the axis was reliably cued before the stimulus appeared, so it is unlikely that regularity discrimination was more difficult in the horizontal condition. It is thus unlikely that task difficulty explains the effect of orientation on alpha ERD. Incidentally, the fact that behavioral discrimination performance was near perfect in all conditions is not relevant here. This was an unspeeded judgment: participants may be correct every time, but still find the discrimination more difficult in one condition than another.

Julesz (1971) suggested that the bilateral symmetry of the visual system made processing vertical symmetric patterns easier than other orientations. Each half of a vertically presented symmetrical pattern is processed via the contralateral cortical hemisphere, with this activation then matched across the vertical midline. This suggests that the corpus callosum mediates the putative advantage of vertical symmetry detection at fixation. Herbert and Humphrey (1996) found support for this callosal hypothesis because two subjects born without a corpus callosum did not detect vertically presented symmetrical patterns quicker than horizontal ones. The effect of orientation on ERD is consistent with the callosal hypothesis in so much as it shows a different neural response when communication across the callosum is required. It is interesting that within-hemisphere connections activated in the horizontal condition produced more alpha ERD than between-hemispheric ones, because shorter connections lead to higher frequency coupling, and greater desynchronization at lower frequencies (Buzsáki, 2006). However, the effect of orientation on ERD should be treated cautiously, because it was highly dependent on preprocessing stages. (There was no ERD difference between horizontal and vertical conditions when the analysis was run without ICA, see supporting information Figure S2).

The SPN was also present in this EEG data: Amplitude was lower in the reflection conditions than the translation conditions from around 250 ms until the end of the epoch. This is similar to what was reported in Makin et al. (2013, 2014). However, the current work makes the novel contribution of showing that the SPN is comparable whether patterns are oriented vertically or horizontally. Previous work by Beh and Latimer (1997) also compared ERPs for horizontal and vertical symmetry; however, they did not show a clear SPN component and their experiment only had a small number of participants, so it is difficult to relate these results to the growing SPN literature on symmetry perception (Höfel & Jacobsen, 2007a; Jacobsen & Höfel, 2003; Makin, Wilton et al., 2012; Norcia et al., 2002).

Another novel finding was that the SPN was more pronounced in right hemisphere electrode clusters. However, this result should be treated cautiously because the crucial Hemisphere × Regularity interaction was eliminated when we adopted different data preprocessing procedures (see supporting information). Nevertheless, the SPN and ERD are both potentially generated by right-lateralized networks, and these signals reflect different aspects of the same or overlapping systems.

The topography and latency of the SPN may be familiar to ERP researchers. Specifically, there are links with the negative-deflection mask, reported by Verlerger, Gorgen, and Jaskowski (2005), but more generally, many ERPs are characterized by a sustained, late wave following the high frequency visual evoked potential (Luck, 2005). For instance, a sustained posterior contralateral negativity is found when people attend to the right or left side of space (Lefebvre, Dell'acqua, Roelfsema, & Jolicoeur, 2011), or when people hold multiple items in visual working memory (Vogel & Machazawa, 2004). Furthermore, presentation of recognizable objects compared to scrambled objects produces a negative late component at posterior electrodes, beginning around 230 ms (Gruber & Müller, 2005; Martinovic, Mordal, & Wuerger, 2011). Of course, different neurocognitive processes generate these ERPs, despite some crude waveform similarity. In summary, the regularity-related SPN is partly defined by the stimuli that produce it, not just latency and topography, which are partly shared with other slow negatives related to visual, motor, attentional, and working memory processes.

### Conclusions

This study has confirmed the presence of a right-lateralized posterior alpha desynchronization during a regularity discrimination task. Previous work has shown that this ERD response is present across all trials. We tested whether the right lateralization was due to a temporary shift of spatial attention to the left, prioritizing contralateral inputs, or to a functional specialization of the right hemisphere for regularity discrimination. If ERD lateralization was produced by participants shifting spatial attention to one side of the pattern, it would disappear when the pattern was oriented horizontally (as moving attention to the right or left would serve no purpose in comparing the two halves). It was found that right lateralization of ERD was equivalent for both orientations. The right bias may therefore reflect specialization of the right hemisphere for regularity discrimination, possibly because the task requires the processing of complex spatial information.

Let us summarize the mixed evidence for right lateralization during regularity discrimination: (a) Psychophysical and neuropsychological work has shown that symmetrical patterns presented to the right hemisphere are detected more quickly. (b) Right hemisphere brain damage has a more profound effect on line bisection. (c) There is no evidence for right lateralization from fMRI. (d) TMS work shows that the right LOC plays a greater role than the left in symmetry discrimination. (e) Alpha ERD is often right lateralized, in all conditions (reflection, random, or translation) and independently of orientation. This response usually occurs when the task is to classify regularity, but not during figure-ground discrimination. (f) The symmetry-related SPN is sometimes weakly right lateralized. (g) There is no comparable evidence for left lateralization. What firm conclusions can be drawn from this mixed evidence? We propose that symmetry perception is bilateral, mediated by extrastriate areas and the LOC, but that the right LOC plays a more prominent role. Although the right lateralization of symmetry discrimination networks is not detected with all neuroimaging techniques under all circumstances, it is likely to be a real phenomenon.

## Supporting Information

**Appendix S1:** Consequences of different data selection procedures and analysis without ICA.

**Figure S1:** Event-related potentials without ICA analysis.

**Figure S2:** Event-related desynchronization without ICA analysis.
