# Supplementary material for: Right-lateralized alpha desynchronization during regularity discrimination: Hemispheric specialization or directed spatial attention?
Source: Psychophysiology. 2014 Dec 23;52(5):638–47. doi: 10.1111/psyp.12399 (PMC4681321; doi:10.1111/psyp.12399)
Supplement: Supplementary file 1 — Appendix S1: Consequences of different data selection procedures and analysis without ICA. [file psyp0052-0638-sd1.doc]

**Supplementary Materials**

**Consequences of different data selection procedures**

In EEG research there is a potential problem with post hoc selection of channels, time windows and frequency bands. This can make statistical procedures invalid, or demand excessively strict multiple comparison correction procedures which would increase the chance of making a Type 2 error. To avoid this, it is advisable to choose parameters a-priori, and this approach is plausible when previous work provides clear effects that can be re examined.

This work replicated the findings of Makin et al. (2014), who also reported ERPs and ERD during a task where participants discriminated reflection from translation patterns. However, the parameters used in that analysis were not quite appropriate for the current data set. There is a tension between the desirability of a priori data selection criteria on the one hand, and capturing effects of interest on the other. This was resolved as follows: In the main manuscript, data was selected to capture the effects that were apparent in the Figures. However, to ensure that the results were not too dependent on these decisions, we re- ran the analysis using the same parameters as Makin et al. (2014).

The SPN results were very similar when PO7 and PO8 electrodes were used, as in Makin et al. (2014). Amplitude was explored in the 250 to 1000 ms window with repeated measures ANOVA (Regularity [Reflection, Translation] X Orientation [Horizontal, Vertical]). There was a main effect for Regularity (F (1,23) = 27.50, p < 0.001, partial *η*^2^ = 0.545). There was no effect of Orientation (F (1,23) = 1.64, p = 0.213, partial *η*^2^ = 0.066) or Regularity x Orientation interaction (F (1,23) = 2.69, p = 0.114, partial *η*^2^ = 0.105). This replicates the main analysis, and confirms that the SPN was not too dependent on the choice of electrodes for analysis.

Next, the ERD analysis was re-run using the same time and frequency bands as Makin et al. (2014), that is, 400-700 ms, 8-13 Hz. There was main effect of Hemisphere (F (1,23) = 9.10, p = 0.006, partial *η*^2^ = 0.284) and Orientation (F (1,23) = 4.96, p = 0.036, partial *η*^2^ = 0.177). There were no other effects or interactions (next largest = Hemisphere X Regularity, F (1,23) = 1.44, p = 0.243, partial *η*^2^ = 0.059). We note, however, that the electrode clusters used here are different to those used by Makin et al. (2014). If the same electrodes were used, the main effect of Hemisphere was no longer significant.

**Analysis without ICA**

This study was designed to re-examine the electrophysiological responses during reflection/translation discrimination reported by Makin et al., (2014) and the ERPs reported by Makin et al. (2013). The pre-processing steps were designed to be as similar as possible to our previous work, to allow the most valid comparison. Nevertheless, it is important to examine potential distortions introduced by the ICA data cleaning procedures. ICA can be used to remove large, unwanted blink and eye movement artifacts, while retaining the cortical responses produced by the resulting visual field changes. To examine this issue, all analyses were rerun *without the ICA cleaning stage*. For SPN, trials were excluded when amplitude exceeded +/- 100 μV during the -200 to 1000 ms window (where all interesting effects were situated). Unsurprising, the mean number of excluded trials increased, from 16% to 30% in this case. For ERD analysis, the exclusion window was -500 to 1000, and 35% of trials were excluded.

SPN amplitude was highly correlated between with and without-ICA versions of the data in every condition (r = 0.82 to 0.96). For SPN, there was still a main effect of regularity (F (1,23) = 35.14, p < .001, partial η2 = 0.604), however the original Regularity X Hemisphere interaction was no longer significant (F (1,23) = 3.28, p = 0.083, partial η2 = 0.125). Supplementary Figure 1 shows the results of the without-ICA analysis in the same format as Figure 2 in the manuscript.

**[Supplementary Figure 1 here]**

For the ERD, there were again correlations between with and without-ICA versions of the data (r = 0.86 to 0.96). The original main effect of Hemisphere was still apparent in the no-ICA analysis (F (1,23) = 5.93, p = 0.023, partial η2 = 0.205), however the main effect of orientation was considerably reduced (F (1,23) = 2.24, p = 0.148, partial η2 = 0.089). This effect therefore can be considered less robust than the other findings of this work. Supplementary Figure 2 parallels original Figure 3.

**[Supplementary Figure 2 here]**
