# Supplementary figures and images for: Right-lateralized alpha desynchronization during regularity discrimination: Hemispheric specialization or directed spatial attention?
Source: Psychophysiology. 2014 Dec 23;52(5):638–47. doi: 10.1111/psyp.12399 (PMC4681321; doi:10.1111/psyp.12399)

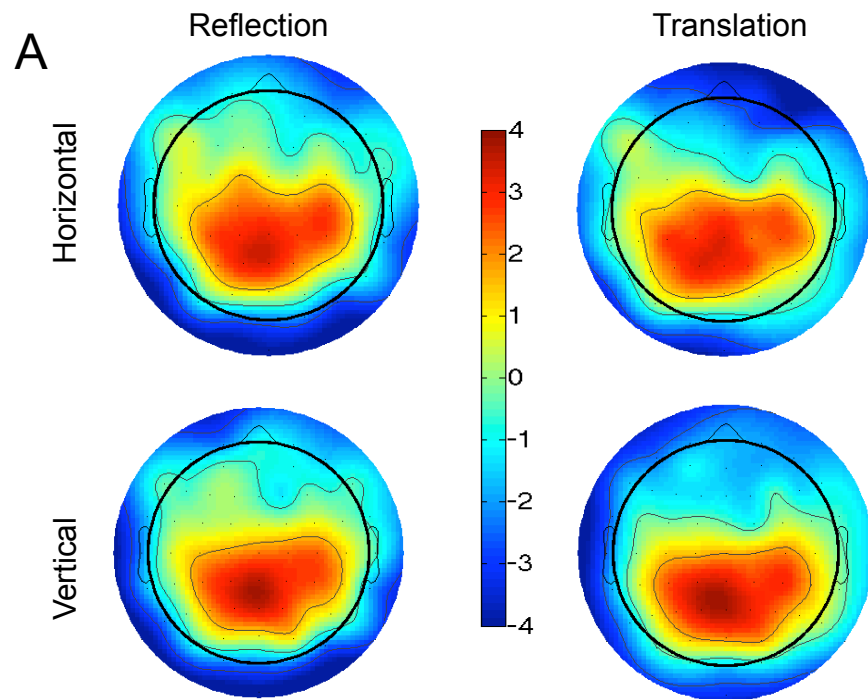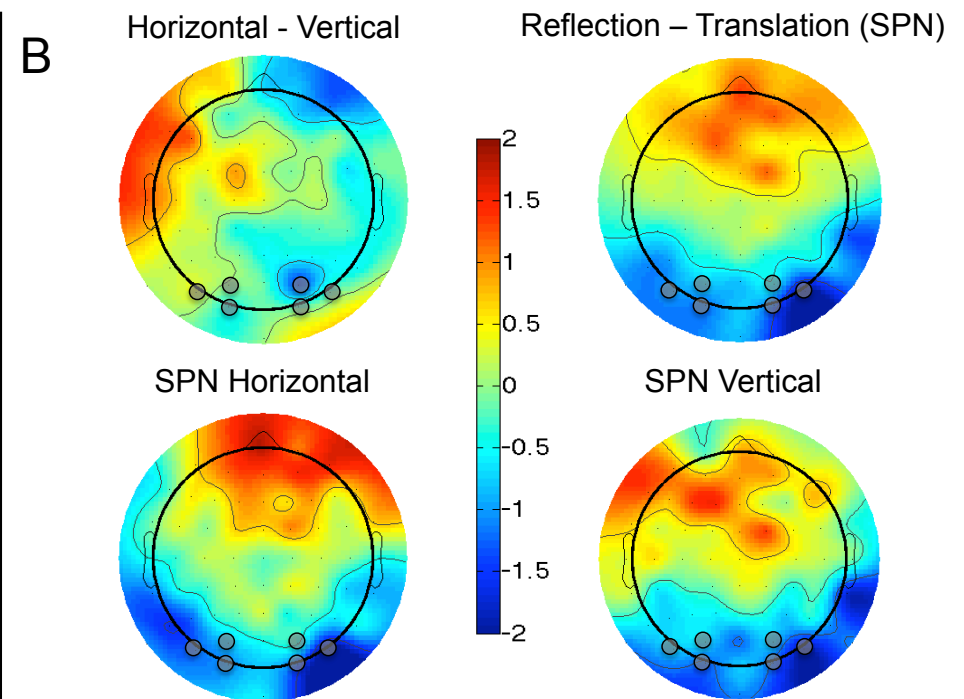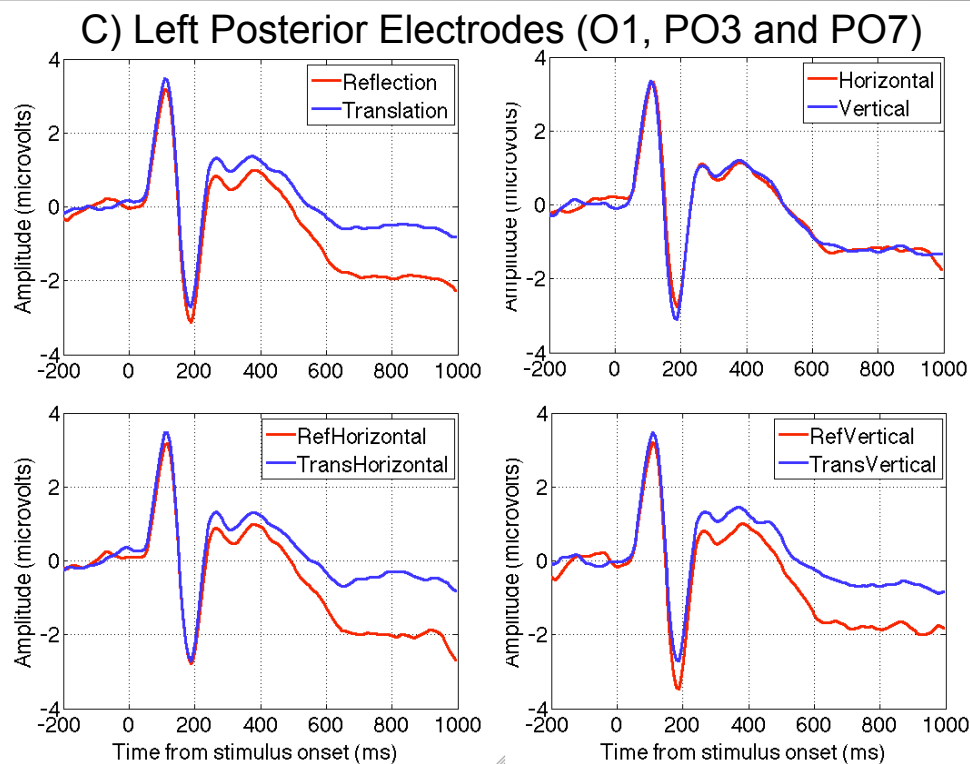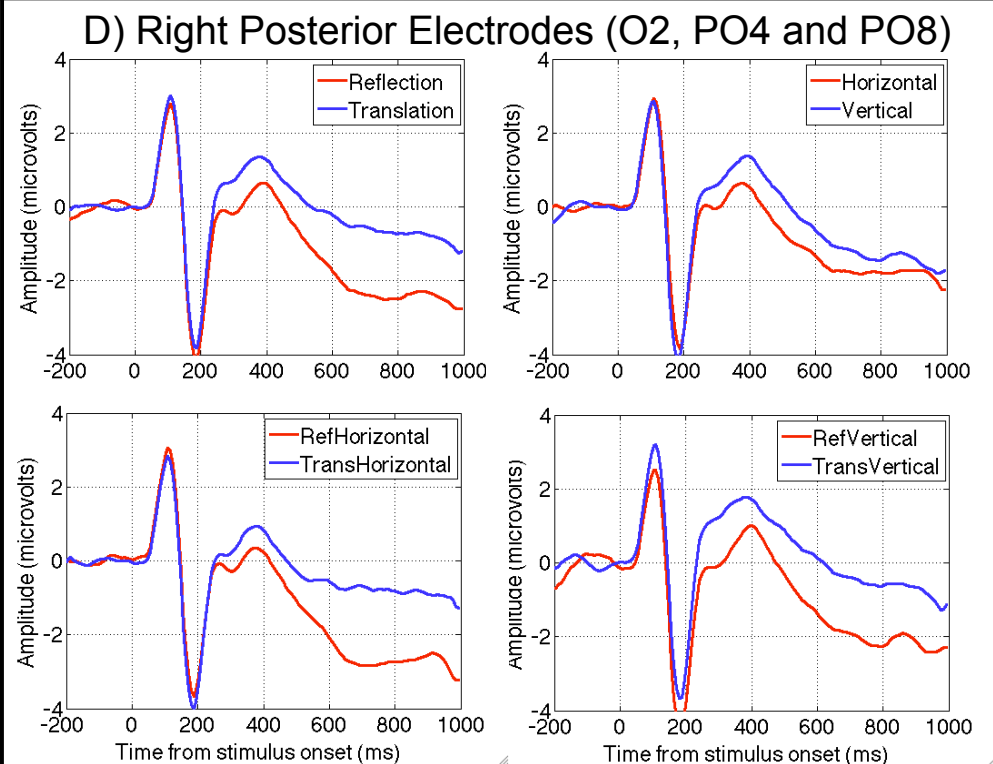

Supplement: Supplementary file 2 — Figure S1: Event-related potentials without ICA analysis. [file psyp0052-0638-sd2.pdf]
