# Supplementary material for: Right-lateralized alpha desynchronization during regularity discrimination: Hemispheric specialization or directed spatial attention?
Source: Psychophysiology. 2014 Dec 23;52(5):638–47. doi: 10.1111/psyp.12399 (PMC4681321; doi:10.1111/psyp.12399)
Supplement: Supplementary file 3 — Figure S2: Event-related desynchronization without ICA analysis. [file psyp0052-0638-sd3.pdf]

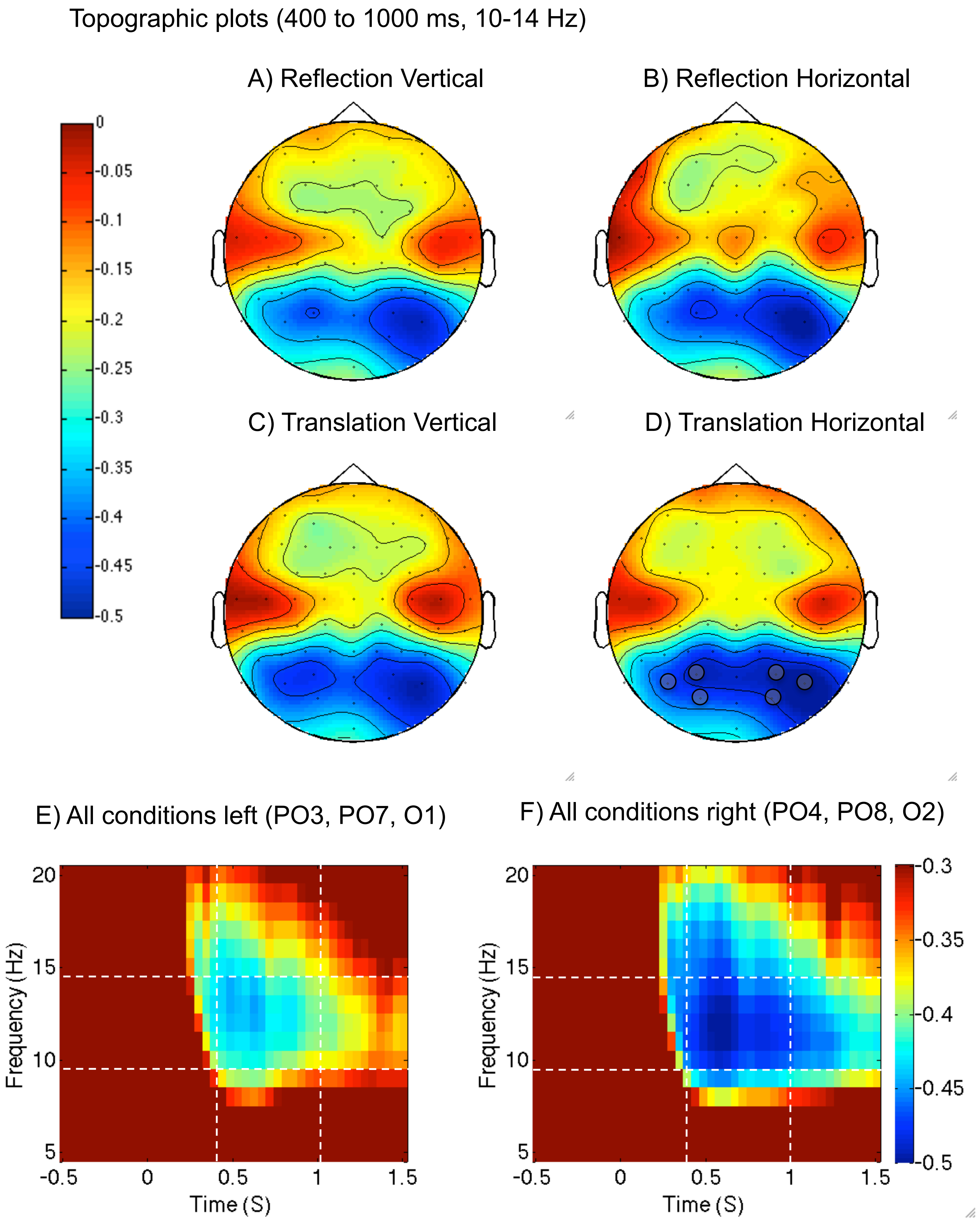

Topographic plots (400 to 1000 ms, 10-14 Hz)
A) Reflection Vertical
B) Reflection Horizontal
C) Translation Vertical
D) Translation Horizontal
0
-0.05
-0.1
-0.15
-0.2
-0.25
-0.3
-0.35
-0.4
-0.45
-0.5
E) All conditions left (PO3, PO7, O1)
F) All conditions right (PO4, PO8, O2)
Frequency (Hz)
20
15
10
5
-0.5
0
0.5
1
1.5
Time (S)
-0.3
-0.35
-0.4
-0.45
-0.5
